# Impact of nickel mining in New Caledonia assessed by compositional data analysis of lichens

**DOI:** 10.1186/s40064-016-3681-4

**Published:** 2016-11-28

**Authors:** Camille Pasquet, Pauline Le Monier, Fabrice Monna, Christophe Durlet, Benjamin Brigaud, Rémi Losno, Carmela Chateau, Christine Laporte-Magoni, Peggy Gunkel-Grillon

**Affiliations:** 1 Pôle Pluridisciplinaire de la Matière et de l’Environnement, Université de la Nouvelle-Calédonie, BP R4, Nouvelle-Calédonie, 98851 Nouméa Cedex, France; 2 UMR 6298, ArTeHis, Université de Bourgogne-Franche Comté, 6 bd Gabriel, Bat. Gabriel, 21000 Dijon, France; 3 UMR 6282, Biogéosciences, Université de Bourgogne-Franche Comté, 6 bd Gabriel, Bat. Gabriel, 21000 Dijon, France; 4 UMR-CNRS 8148, Géosciences Paris-Sud, Université Paris-Sud, Bât. 504, 91405 Orsay Cedex, France; 5 UMR CNRS 7154, Institut de Physique du Globe de Paris (IPGP), Sorbonne Paris Cité, Université Paris Diderot, 1 rue Jussieu, 75013 Paris, France; 6 UFR SVTE, Université de Bourgogne-Franche Comté, 6 bd Gabriel, Bat. Gabriel, 21000 Dijon, France

**Keywords:** Trace metal, Biomonotor, Ultramafic rock

## Abstract

The aim of this study is to explore the use of lichens as biomonitors of the impact of nickel mining and ore treatment on the atmosphere in the New Caledonian archipelago (South Pacific Ocean); both activities emitting also Co, Cr and possibly Fe. Metal contents were analysed in thirty-four epiphytic lichens, collected in the vicinity of the potential sources, and in places free from known historical mining. The highest Ni, Co, and Cr concentrations were, as expected, observed in lichens collected near ore deposits or treatment areas. The elemental composition in the lichens was explored by multivariate analysis, after appropriately transforming the variables (i.e. using compositional data analysis). The sample score of the first principal component (PC1) makes the largest (positive) multiplicative contribution to the log-ratios of metals originating from mining activities (Ni, Cr, Co) divided by Ti. The PC1 scores are used here as a surrogate of pollution levels related to mining and metallurgical activity. They can be viewed as synthetic indicators mapped to provide valuable information for the management and protection of ecosystems or, as a first step, to select locations where air filtration units could be installed, in the future, for air quality monitoring. However, as this approach drastically simplifies the problem, supplying a broadly efficient picture but little detail, recognizing the different sources of contamination may be difficult, more particularly when their chemical differences are subtle. It conveys only relative information: about ratios, not levels, and is therefore recommended as a preliminary step, in combination with close examination of raw concentration levels of lichens. Further validation using conventional air-monitoring by filter units should also prove beneficial.

## Background

Nickel exploitation began in the archipelago of New Caledonia (South Pacific Ocean), soon after the discovery by Jules Garnier in 1864 of nickel silicates, called garnierite. Ever since, the economic life of the island has beaten to the rhythm of nickel demand. Nickel mining is now becoming the largest employer in New Caledonia (more than 7500 employees in 2015). When exploitation first began, high-grade veins of garnierite (6–7% Ni), identifiable by their green colour, were extracted. Miners were soon interested by other lateritic horizons that developed on ultramafic weathered rocks. These nickel-enriched soils are located near the surface, so opencast mining was preferred. For such an approach, vegetation and top soil are first removed, and ore is extracted and transported by trucks on unpaved roads or by belt conveyors to loading zones or to ore treatment plants. All these operations are expected to release into the atmosphere huge amounts of dust enriched in trace metals (Chakraborty et al. 2002; Huertas et al. 2012), known to affect human health: it can lead to respiratory dysfunction, cardiovascular disease and cancer (International Agency for Research on Cancer 1990; Andersen et al. 1996; Harrison and Yin 2000; Menvielle et al. 2003). Nickel mining and ore treatments are known to have deleterious effects on the quality of the New Caledonian environment by degrading natural ecosystems (Bramwell 2011; Kettle et al. 2007; Losfeld et al. 2015), forests (Jaffré et al. 1977), and the lagoon (Gunkel-Grillon et al. 2014; Hédouin et al. 2007). Furthermore, the smallest dust particles can be spread over very long distances, far from emission sources. The impact of mining on the surroundings and the geographical dispersion of metals are often estimated using atmospheric filtration units covering the territory of interest. The filters are frequently changed because meteorological conditions (humidity, wind strength and direction variability, etc.) drastically affect the quality and quantity of dust material collected. This procedure is commonly used as a reference method, but remains costly and time consuming. Yet, at least for preliminary screening, some alternatives do exist. They are based on the biomonitoring capabilities of some organisms or organic material, such as tree rings, peat deposit, mosses or epiphytic lichens (Aslan et al. 2012; Balabanova et al. 2013a; Boamponsem et al. 2010; Mihaljevič et al. 2008; Monna et al. 2012; Richter et al. 2007; Spiro et al. 2004). Epiphytic lichens are symbiotic organisms, composed of fungi and algae. They have the ability to absorb and accumulate metals, through wet and dry deposition, without symptoms, at least up to a certain level (see the seminal work from Nylander 1866, and for reviews of recent studies, Conti and Cecchetti 2001; Szczepaniak and Biziuk 2003). Their nutrient uptake therefore relies exclusively on air constituents, since they have neither roots nor cuticles. Concerning absorption of metals, three main mechanisms have been invoked: intercellular absorption by an exchange process, intercellular accumulation, and entrapment of metal-rich particles (Richardson 1995; Szczepaniak and Biziuk 2003). The respective role of these processes is, however, not fully understood, all the more since metal contents in thalli seem to experience periods both of accumulation and release, related to several environmental factors. In any case, it has been demonstrated that lichens are good biomonitors of trace elements, as their thalli concentrations are correlated with those in their surrounding environment (Conti 2008). The sampling of these organisms is inexpensive and easy (when thalli are abundant), in any case free from heavy logistics. Furthermore, it can be problematic to compare concentrations from different individuals, even within the same species, because biomonitors may be affected by several internal factors, such as body morphology, age, and exposure (Carignan et al. 2002; Doucet and Carignan 2001; Kinalioglu et al. 2010; Senhou et al. 2002). That is why, instead of examining absolute concentration levels, some authors have proposed normalizing concentrations of potentially anthropogenic elements by a single chosen reference element, exclusively of crustal origin, such as titanium, aluminium or a lanthanide (i.e. all metal concentrations are divided, for instance, by those of Ti, the normalizing element used in the following, Goix et al. 2013; Monna et al. 2012). This can be viewed as the first step towards the so-called enrichment factor (EF) calculation, which aims at detecting, in combination with other tools, which elements are enriched in relation to local soils or earth crust (Agnan et al. 2013; Aničić et al. 2009; Aslan et al. 2012; Cloquet et al. 2015). The only difference is that there is no final normalization by local reference values or, more simply, by those of the upper continental crust (e.g. Wedepohl 1995).

Although convenient, and most of the time efficient (Bergamaschi et al. 2002; Nyarko et al. 2006), the normalization of concentrations of potentially anthropogenic elements by a single chosen reference element (e.g. Metals/Ti or Metals/Al), or possibly by the sum of reference elements (e.g. Metals/(Ti + Al)), presents an unavoidable drawback: the use of new ratio-based variables with the same element(s) as denominator is mathematically inadequate for all statistics based on correlations (see Pearson 1896; Kim 1999, for a complete discussion, and Monna et al. 2006 for a debatable use of multivariate statistics on such ratio data). The use of [metal/Ti] or [metal/(Ti + Al)] ratios are still mathematically correct when correlations are not computed (only sites are compared), but the geochemical signals carried by all other pairwise ratios (e.g. Ni/Cr, Co/Zn, etc.) are not really taken into account, although they may contain subtle information about the origin of the metals.

 The compositional data are by nature closed because the sum of all components, including those not measured, is constrained (to 1, or to 100%). The covariance structure of such a dataset is necessarily biased (Filzmoser et al. 2009a, b), so that most multivariate techniques become doubtful without a proper transformation. Undeniable progress has, however, been made since the 1980s to open the dataset and to remove problems related to spurious inter-element correlations (Aitchison 1982; Van der Weijden 2002). The data are, with these new techniques, treated as a whole, allowing each inter-elemental ratio to be examined (Van den Boogaart and Tolosana-Delgado 2013).

In the present study, elemental concentrations in Co, Cr, Ni, Fe, Cu, Zn and Ti were analysed in more than 30 epiphytic lichens collected in New Caledonia, at various distances from modern mining activities, and from an ore treatment plant in Noumea, from within the city, and from areas free from any known historical mining. Ti is supposedly of crustal origin and used as reference, Cu and Zn have potentially an industrial/domestic origin, while Ni, Co, Cr and, to a lesser extent, Fe are especially targeted, because they are expected to be emitted by mining and metallurgical activity. The objective of this study is therefore to evaluate the potential of lichens as biomonitors of air quality in the context of Ni mining and ore treatment. Compositional data analyses were used to compute a synthetic index summarising the local degree of contamination related to mining and metallurgy.

## Methods

### Site area

**Fig. 1:**
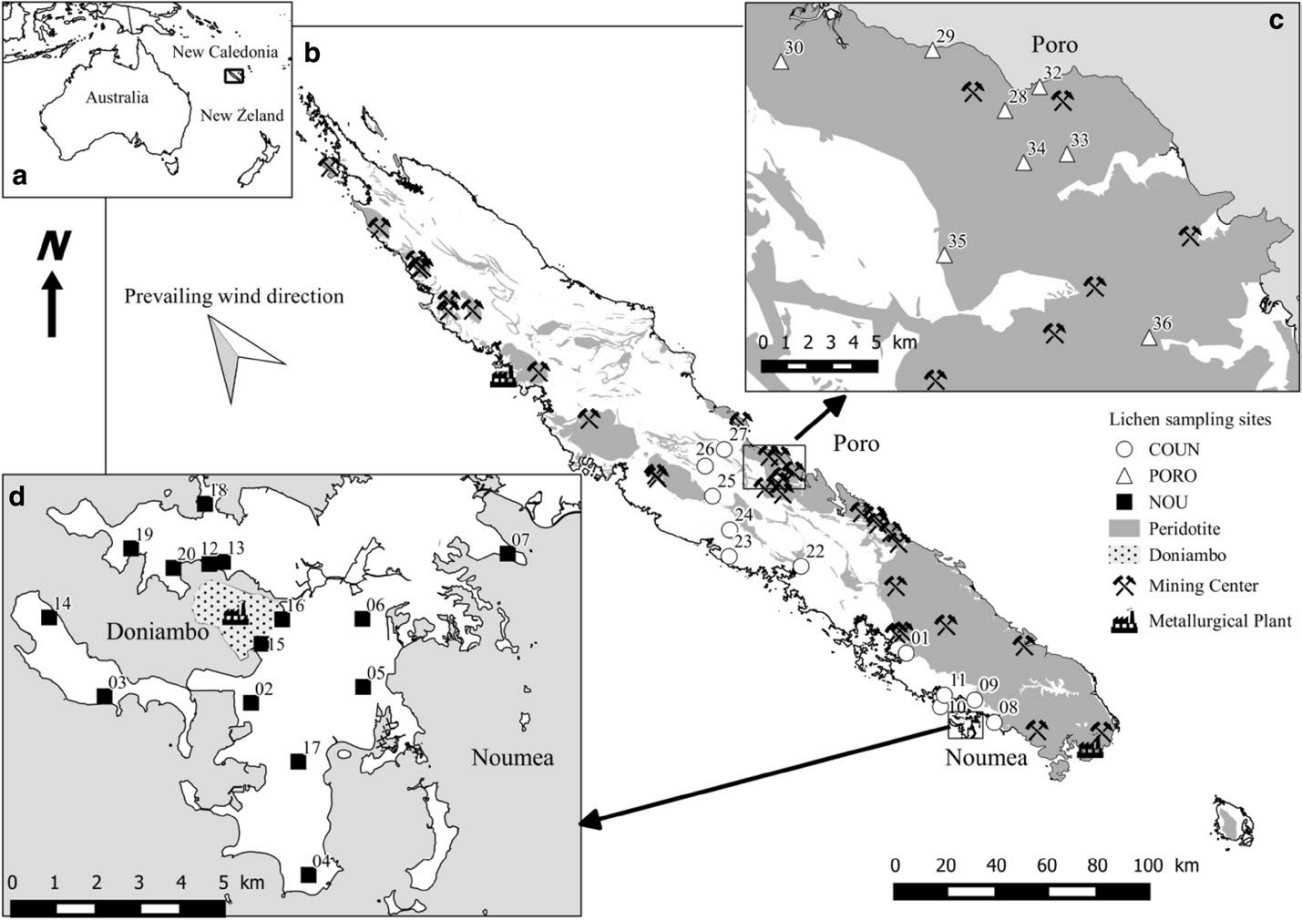
**a** Location of New Caledonia in Pacific Ocean, **b** map of New Caledonia, **c** close up on the Poro mine region, situated in the East Coast, and **d** close up on Noumea. Sampling sites are provided with identification numbers

New Caledonia is an archipelago located in the South Pacific Ocean, 1300 km east of Australia (Fig. 1a). The climate is semitropical, characterized by a warm rainy season and a cooler season. Annual mean rainfall on the main island, “Grande Terre”, is 1700 mm for a mean temperature of 25 °C, with considerable differences between the east and west coasts. The prevailing wind blows from the south-east all year long. Grande Terre is more than 400 km long and 50 km wide (Fig. 1b). Two-thirds of this area is composed of weathered ultramafic rocks, mainly peridotites, forming lateritic and saprolitic horizons (Chevillotte et al. 2006). These horizons are enriched in transition metals, such as nickel, chromium, cobalt and manganese (Trescases 1973). These metals are extracted from about thirty large, active open-pit mines. New Caledonia accounted in 2011 for about 2% of the worldwide production of Co and 8% of Ni (Wacaster 2012). Chromium was also mined from 1880 to 1962, producing during this period even more metal than Ni. After a decade of decline, the Cr production experienced a revival in 1976, with the reopening of Cr mines which targeted chromite. As key insights, the New Caledonian laterites represent massive reserves of iron. Some of them were mined between the 1930s and the late 1960s.

Two sites directly related to nickel mining and smelting were more particularly targeted in this study. The first is the Poro mine, situated on the east coast, one of the oldest still active extraction sites (Fig. 1c). The second is the metallurgical ore factory of Doniambo, located in Noumea (Fig. 1d). It was established in 1910 on an isolated, 7 ha site, far away from the town of Noumea in its past configuration. Today, this site covers 250 ha. It is surrounded by many industrial areas, the Noumea harbour and, more problematically, several residential zones set up following the considerable extension of the regional capital, Noumea, which now counts 100,000 inhabitants. About 3.5 million tons of ores per year, extracted from several mines all over Grande Terre, and transported by tankers, are treated in this pyrometallurgical plant. The proximity of the Doniambo plant to the capital generates environmental atmospheric issues for residents, in terms of (i) SO_2_ levels, a pollutant emitted from the fuel power plant powering the energy-consuming pyrometallurgical operations, and (ii) nickel-enriched PM_10_ (particulate matter with a diameter below 10 µm) released from the plant. The situation is such that atmospheric pollution levels are given with the TV weather forecast.

### Sampling

Thirty-four epiphytic lichen thalli belonging to the Parmeliaceae family were collected in New Caledonia in March 2012 from around the Poro mine (PORO, n = 8), and the Noumea peninsula (NOU, n = 15). Several additional lichens were also collected from the countryside between these two sites (COUN, n = 11), far from any known historical mining sites or potentially polluting human activities (Fig. 1b–d). Lichens were collected exclusively from tree trunks, always at least 1.5 m above ground level, by means of pre-cleaned plastic knives. Sometimes, lichens were moistened with Milli-Q water to facilitate their removal from the trunks. Any tree bark fragments collected with thalli were systematically eliminated. The samples were immediately stored in hermetically closed plastic bags.

### Chemical procedure

In the laboratory, lichens were dried at 80 °C overnight, and crushed in a pre-cleaned agate mortar to obtain a fine, homogeneous powder. The sample preparation follows that of Monna et al. (2012). Briefly, about 100 mg of powdered lichen (precisely weighed) was placed in a PTFE beaker (Savilex) with 2 mL each of suprapure HCl, HNO_3_ and HF heated at 105 °C, until complete digestion. Blanks and biological certified reference materials (CRMs): peach leaves (NIST-1547) and lichens (BCR-482) were processed with each batch of unknown samples. Solutions, adequately diluted with Milli-Q water, were analysed for Co, Cr, Ni, Fe, Cu, Zn and Ti, using an Inductively Coupled Plasma-Optical Emission Spectrometry (ICP-OES) ARCOS Spectro, installed in an ISO-5 clean room at the University of Paris Diderot. Limits of detection (LOD) were evaluated using three times the standard deviation of five analytical blanks. Measurements for most elements did not differ by more than 15% from certified values (Table 1).

**Table 1:** Quality control of the analyses

	Co	Cr	Cu	Fe	Ni	Zn	Ti
LOD (µg g^−1^)	0.2	0.2	0.2	2	0.2	0.1	0.6
BCR 482 (µg g^−1^)							
Measured	0.5	3.3	6.2	701	2.3	87	78
Certified	0.32^a^	4.12	7.0	804^a^	2.5	100	–
NIST 1547 (µg g^−1^)							
Measured	<LOD	1.0	2.6	185	0.8	17.4	23
Certified	0.07^a^	1.0^a^	3.7	218	0.69	17.9	23^b^

LOD (limit of detection) is provided considering the dilution factor, so that LOD can be compared to the raw sample concentrations; BCR 482 is a lichen standard provided by the Institute for Reference Materials and Measurements; NIST 1547 is a peach leaves standard provided by the National Institute of Standards and Technology

– No data available

^a^Provided but not certified

^b^Value from Monna et al. (2012)

The replicability of lichen measurements was tested with five samples processed twice, independently (Table 2). Variations between replicates can be higher than the deviations observed for CRMs, probably because natural material, even finely crushed, remains more heterogeneous than CRMs. However, these deviations are unimportant in comparison with the concentration ranges observed at the scale of the entire study (Table 2).

**Table 2:** Body metal concentrations in lichens (in µg g^−1^, except iron content in %w/w)

Sample ID	Groups	Co	Cu	Cr	Fe	Ni	Zn	Ti
1	COUN (countryside)	2.1	8.2	26	0.19	43	75	128
8		2.0	2.2	18	0.24	26	59	181
9		2.0	3.5	19	0.17	83	30	100
10		21	19	120	2.12	443	92	2045
11		11	21	61	3.29	75	52	3974
11bis		10	16	58	3.23	65	45	3669
22		3.4	0.82	20	0.53	32	76	471
23		2.1	2.9	23	0.19	28	77	163
23bis		1.4	8.5	21	0.15	23	68	137
24		6.6	21.4	30	0.75	60	20	478
25		3.2	0.63	29	0.23	54	89	172
26		5.0	6.4	58	0.37	51	23	347
27		0.85	0.63	13	0.10	20	20	73
Geom mean	NOU (Noumea)	3.5	3.9	30	0.39	52	47	311
2		26	38	133	0.75	822	87	299
3		2.8	5.6	14	0.22	64	28	195
4		0.84	0.63	4.8	0.04	24	33	32
5		3.9	3.8	24	0.17	130	103	123
6		1.8	4.8	13	0.08	64	29	33
7		2.4	0.63	18	0.11	66	67	60
12		15	21	51	0.80	238	77	761
13		53	9.2	346	2.02	1981	934	939
14		12	2.4	60	0.45	429	69	188
15		100	15	319	2.15	4140	153	506
16		130	37	1046	4.67	4536	259	1138
16bis		124	23	966	4.35	4246	243	1033
17		15	13	86	0.92	427	110	711
18		12	8.4	76	0.49	432	63	226
19		50	111	400	2.16	1645	238	908
19bis		42	91	318	1.85	1462	230	696
20		33	9.2	157	0.94	1344	99	201
Geom mean	PORO (Poro)	13	7.7	72	0.52	400	95	247
28		176	2.4	1612	6.68	3536	147	64
29		14	0.47	111	0.53	204	33	10
30		24	5	254	1.37	391	36	164
32		323	15	1503	6.15	5216	76	69
32bis		294	14	1262	6.48	4725	67	61
33		11	6	666	1.84	325	30	43
34		0.31	0.70	12	0.04	12	3	3
35		11	0.76	118	0.48	191	49	28
36		64	13	565	2.02	949	44	28
Geom mean		21	3.7	272	1.12	418	35	30

The geometric mean is also calculated for each group (the nth root of the product of n values). bis: independent replicates

### Data treatments

**Fig. 2:**
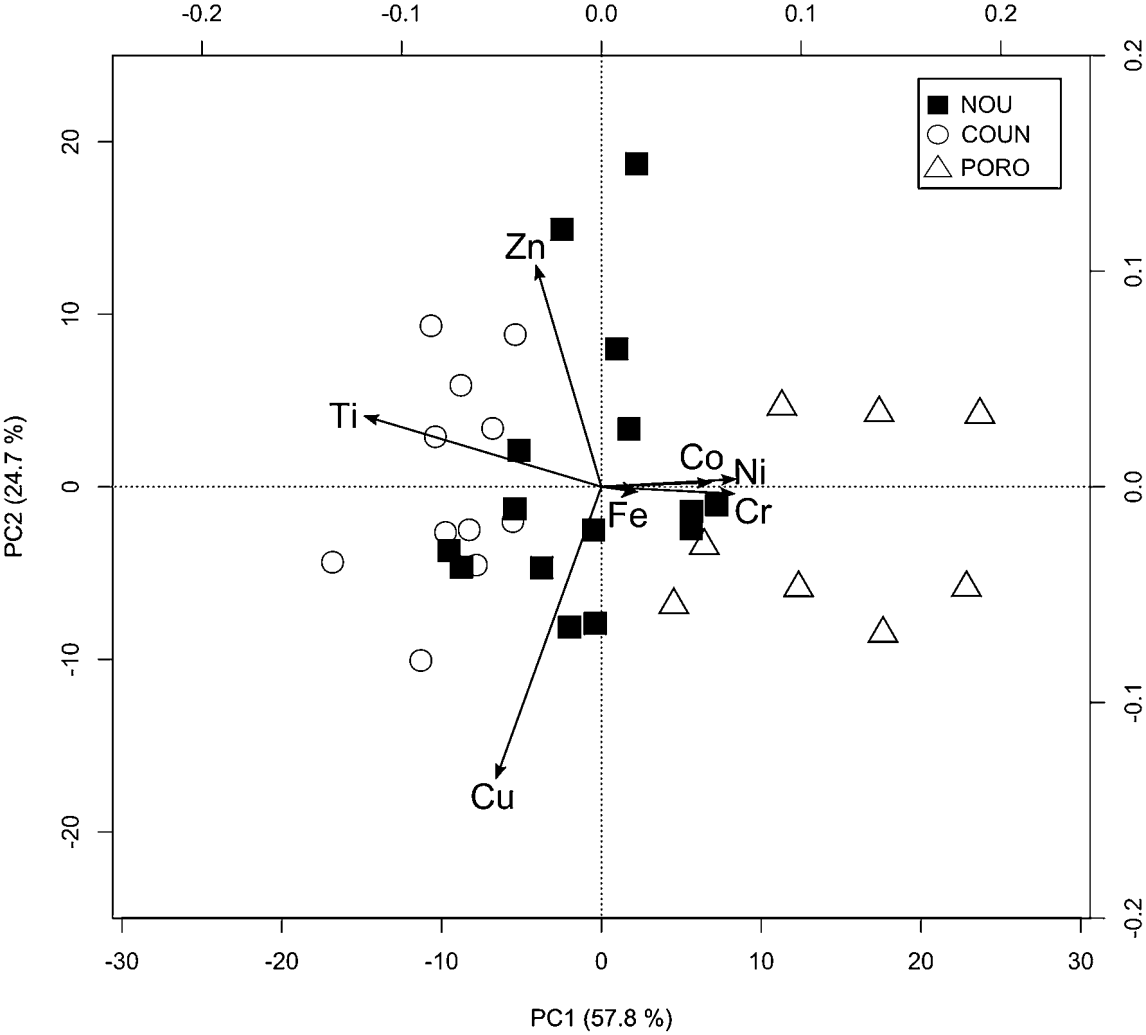
Compositional biplot for lichens projected on the first two principal components

Basically, the elemental composition of a sample is defined by a (raw) vector, composed of D parts, corresponding to the subset of the D concentrations measured $: x = \left[ {x_{1} ,x_{2} , \ldots ,x_{D} } \right]$ (D = 7 in our case, for Ni, Cr, Co, Fe, Zn, Cu and Ti). A centred log-ratio transformation, *clr*(.), was applied to these raw concentrations to circumvent problems related to the closed nature of such data (Aitchison 1982, 1986):

$$clr\left( \varvec{x} \right) = \left[ {\ln \frac{{x_{1} }}{{g_{m} \left( \varvec{x} \right)}},\ln \frac{{x_{2} }}{{g_{m} \left( \varvec{x} \right)}}, \ldots ,\ln \frac{{x_{D} }}{{g_{m} \left( \varvec{x} \right)}}} \right],$$

where g_m_(**x**) corresponds to the geometric mean of the parts of the compositional vector, $g_{m} \left( \varvec{x} \right) = \left( {x_{1} .x_{2} \ldots x_{D} } \right)^{1/D}$. Such a transformation is known to remove the closure constraint, making it usable in Principal Component Analysis (PCA), which is computed to summarise the structure of the data in a lower dimensional space. From this new set of coordinates, the biplot was built, first by singular value decomposition, and then by calculating the biplot coordinates. In this representation, both variables and samples are projected on the same 2D plot. Among the different constructions possible, that favouring the covariance structure of variables over the position of individuals was preferred (Fig. 2, see Aitchison and Greenacre 2002; Daunis-i-Estadella et al. 2011 for more details). It should be noticed that the compositions are now recognized as providing information only on the relative magnitude of their components (Faith 2015). This means that interpretations made from the compositional biplot are, by nature, drawn from ratios between all components, and not from individual components taken separately, as with the classical biplot. Computing a multivariate analysis of variance (MANOVA) on *clr*-transformed data is not possible, because the rows of this matrix sum up to 0, so that the covariance matrix is singular. An alternative consists in using isometric log-ratio transformed data as input matrix, as proposed by Egozcue et al. (2003):

$$ilr\left( x \right) = z = \left[ {z_{1} , \ldots ,z_{D - 1} } \right] \in {\mathbb{R}}^{D - 1} ;z_{i} = \sqrt {\frac{i}{i + 1}} \ln \sqrt[i]{{\frac{{\mathop \prod \nolimits_{j = 1}^{i} x_{j} }}{{x_{i + 1} }}}}\quad {\text{for}}\quad i = 1, \ldots , \, D - 1$$

Once it has been demonstrated that there is a difference between at least one pair of group population means, multiple comparisons can be performed using Hotelling T^2^ tests, along with Bonferroni corrections, as usual.

Data preparation, transformations, statistical procedure and plot drawings used the free R software (R Core Team 2008), with the FactoMineR (Lê et al. 2008), compositions (Van den Boogaart and Tolosana-Delgado 2008), hotteling (Curran 2013) and ggplot2 packages (Wickham 2009). Mapping used the Quantum GIS free software (QGIS Development Team 2010).

## Results and discussion

### Elemental concentrations in lichens

Concentrations in lichens, together with the geometric means of the three groups (NOU, PORO and COUN), are reported in Table 2. They vary widely, covering 2 or 3 orders of magnitude. Such huge variability is rather unusual in environmental studies dealing with lichens, where differences of one or two orders of magnitude are more common (Goix et al. 2013; Zvěřina et al. 2014). As expected, the highest Co, Cr and Ni concentrations (above 100, 1000 and 3000 µg g^−1^, respectively) are observed in two of the Poro samples (PORO #28 and #32), and close to the Doniambo plant (NOU #16) (Table 2). Maximum Zn and Cu contents are recorded at Noumea (NOU #13 and #19). For comparison, the Co, Cr, and Ni concentrations reported for lichens collected close to an open-pit olivine mine in Greenland (Søndergaard 2013) were about one or two orders of magnitude lower than those observed at Poro, but comparable with our COUN samples. Although, at first sight, the overall picture drawn from absolute concentrations seems to be reliable, a non-negligible part of the variations observed in the Co, Cr and Ni concentrations might also be due to body morphology, age, and exposure of individuals, as suggested by the huge variations in Ti contents, an element assumed of crustal origin. Rather than normalizing all metals to a single crustal element (e.g. Ti), and hence comparing the sites, a compositional data analysis approach was preferred here.

### Compositional data analysis

The first two axes of the covariance biplot computed after a *clr*-transformation of the dataset explain 82.5% of the total variability (57.8 and 24.7% respectively), a value reasonably high for D = 7 parts (Fig. 2). This representation must, however, be interpreted differently from the traditional biplot originally proposed by Gabriel (1971), because all the components are somehow intermixed during the *clr*-transformation. Its key reading is the link between two variable arrow heads, which approximates the standard deviation of their corresponding log-ratios (see Aitchison and Greenacre 2002; Van den Boogaart and Tolosana-Delgado 2013 for more details about compositional biplots). The mining elements (Ni, Co, Cr), and to a lesser extent Fe, plot relatively close to each other (i.e. their links are short). They therefore present relatively constant log-ratios, while the largest links observed between [Ni, Co, Cr] and Ti, on the one hand, and between Zn and Cu, on the other hand, indicate the most relative variations across the lichens. Interestingly, the orthogonality between these links underlines the absence of correlation between their corresponding log-ratios (e.g. Zn/Cu is not correlated with Ni/Ti).

**Fig. 3:**
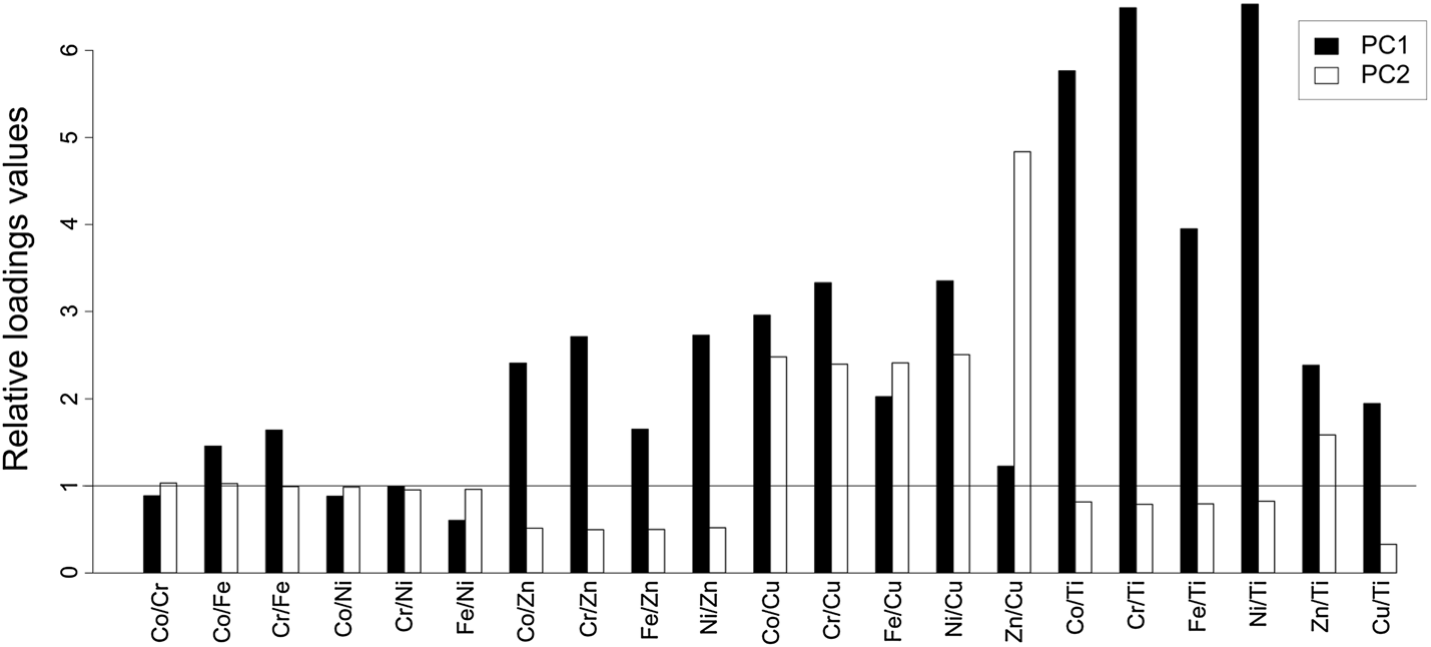
Barplots of loadings of compositional principal component analysis

The influence of the principal components (PCs) on each pairwise ratio can also be displayed by a barplot, where bars represent ratio loadings on PCs. Bar heights above (or below) the horizontal line y = 1 denote a positive (or negative) multiplicative influence on the PCs concerned (Fig. 3, see Daunis-i-Estadella et al. 2011 for mathematical construction). Thus, a loading equal to 1 in the *clr*-transformed space acts somewhat similarly to a zero loading for common PCA. In our case, PC1 makes the largest (positive) multiplicative contribution to Co/Ti, Cr/Ti, Ni/Ti, and to some extent Fe/Ti, while PC2 mostly acts on Zn/Cu.

The three previously defined groups of lichens can be distinguished mainly by PC1 (Fig. 2). The PORO samples plot on the right of the diagram (positive side of PC1), while COUN samples plot on the left (negative side of PC1). The NOU samples lie between the two other groups. Although this structure is clearly visible, it worth noting that multivariate analysis of variance (MANOVA) can also be applied in less trivial cases. The MANOVA indicates that at least one group appears to be significantly different from the others (*p* < 10^−6^). Multiple pairwise Hotelling T^2^ tests indicate that each group is significantly different from the others (*p* < 10^−4^), even after Bonferroni correction (Holm 1979).

### Impact levels and geographical dispersion

**Fig. 4:**
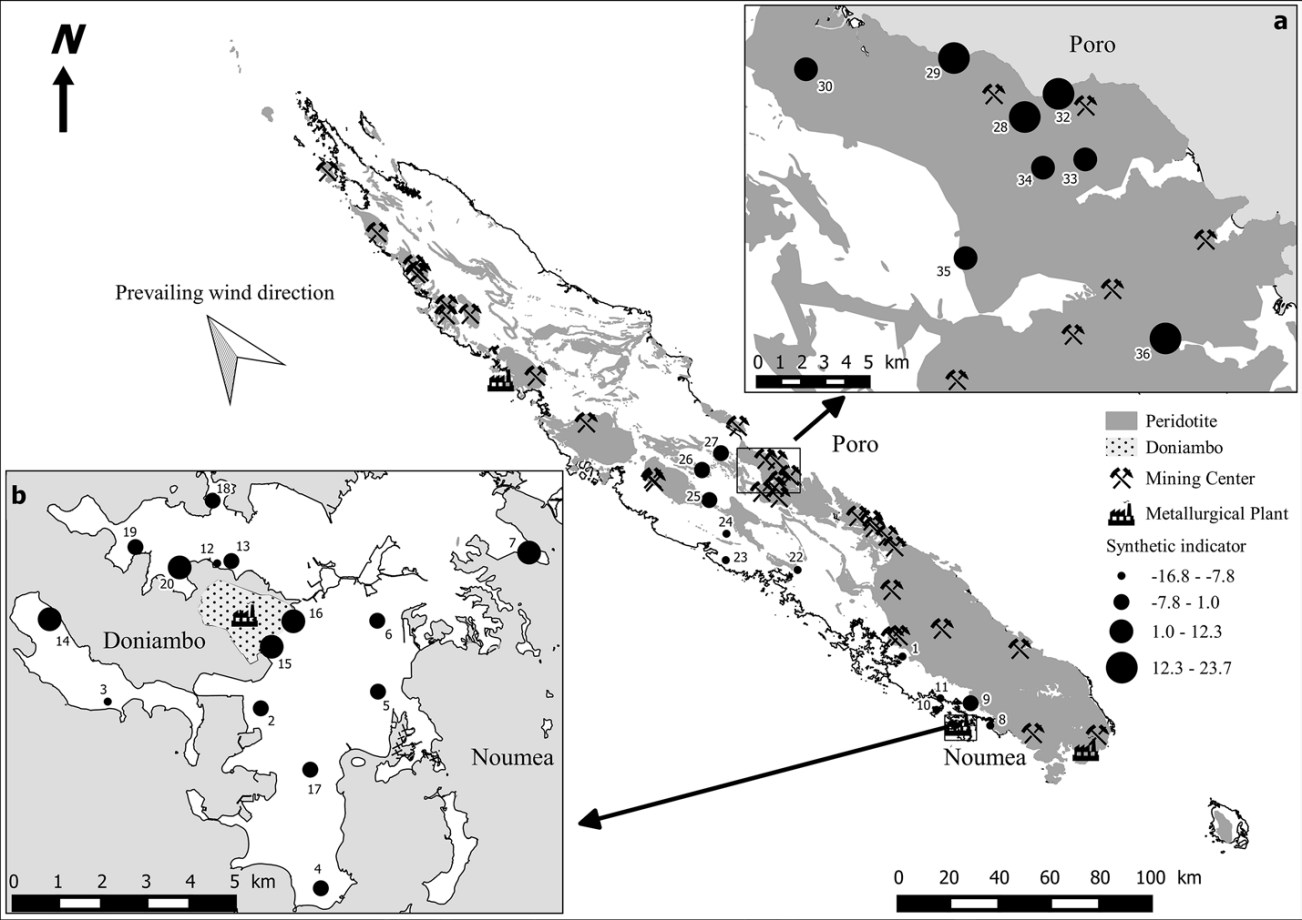
Synthetic indicator of lichen pollution, **a** in Poro and **b** in Noumea

Several multi-element indices have previously been constructed to evaluate the overall quality of a given environment with regard to metal contents (Chen et al. 2005; Holy et al. 2009; Marvin et al. 2004; Nimis et al. 2000). These methods often used threshold and probable effect levels (TELs and PELs), which represent the concentrations above which adverse biological effects are expected to occur, either rarely or frequently. Other methods use mean concentrations of accumulated elements in lichens (Boamponsem et al. 2010; Nimis et al. 2000) or ranks based on percentiles (Fliedner et al. 2014; Holy et al. 2009; Schröder and Pesch 2010). They can be enriched with environmental information, leading to the establishment of specific ecoregions (Schröder and Pesch 2007). Factors like altitude, soil texture, precipitation, or global radiation make it more difficult to compare territories that are very different from in geography. Another common practice consists in treating chemical compositions as a whole. First, a Factor Analysis or a Principal Component Analysis is computed on normalised variables, then a varimax rotation can be operated, producing varifactors (Varol 2011; Zhuang and Gao 2014). This last step seeks to produce strong correlations between the new factor loadings and certain variables of interest, and low correlations with the others, thus leading to a clearer interpretation of the resulting factors (Reimann et al. 2002). The final objective is, if possible, to isolate a factor coinciding with the main pollutants, so that the score of a sample on this axis can be used as an integrated index of the contamination level (Balabanova et al. 2013b; Cicchella et al. 2014). The problem with such a procedure is that, except for some studies (e.g. Filzmoser et al. 2009b), the multivariate analysis is almost never computed using appropriate transformations, specifically designed for compositional data. Meyer et al. (2015) recently proposed a method based on the scores of the compositional biplot, computed after a simple *clr*-transformation of the original variables. In our case, PC1 has a strong positive multiplicative contribution to the log-ratios of metals originating from mining activities (Ni, Cr, Co)/Ti. As a consequence, the varimax rotation is unnecessary. Examining PC1 is somewhat similar to the traditional approach, which consists in considering the metal/Ti ratios separately (rather than the raw concentrations) in order to reduce blurring effects due to lichen exposure, age or species (Dongarrà et al. 1995; Monna et al. 2006). The main difference is that, in our case, all metals of interest are processed together, and integrated into the PC1 sample scores. These values can then be directly used as a synthetic contamination index, at least for a group of elements: Cr, Ni and Co, in other words the elements of interest, emitted into the atmosphere by mining and metallurgical activity. Figure 4, which depicts the individual scores, exhibits a clear spatial structure. The atmosphere around the Poro mine and in the city of Noumea is unambiguously enriched in Ni, Cr, and Co compared to areas free or far from mines. This enrichment is greater at Poro than in Noumea. Although the sampling network was neither dense nor regular, it seems reasonable to estimate that a distance at least 5 km from pollution sources is necessary to return to ‘clean’ conditions. This estimate is similar to those reported in two studies based on lichens: Cloquet et al. (2006) for Metz, a French city, and Søndergaard (2013) for a mining context in Greenland. No clear influence of prevailing winds was noticed in our context, probably because of the sparse sampling scheme, in contrast with a study at Agadir, where strong, persistent sea winds scatter the pollutants up to about 25 km eastwards (Monna et al. 2012). At this stage, it should be noted that our synthetic indicator was designed to facilitate the understanding of a complex process, here the impact of Ni mining and metallurgy on the atmosphere. Although it succeeds in this task, making clearer the overall picture of the aerial dispersion around the main sources, it is not able to take into account the small differences in terms of chemical compositions that might exist between the principal sources.

The PC2 sample scores, which mainly depict the log ratio of Zn/Cu, are not very informative here, because the three groups of samples (NOU, COUN and PORO) do not exhibit any significant difference concerning this variable, neither in terms of mean, nor in terms of variance (Fig. 2).

Although the results are fully coherent in their present form, the addition (or suppression) of elements may drastically modify the outputs of the multivariate analysis. The environmental question must therefore always be kept in mind, so that only those elements supposed to be of interest are processed; the sought-after signals might otherwise be obscured. The transformations required to process the compositional data properly mean that the original concentration values are no longer directly present. This loss of contact can be a serious problem in certain studies, such as those undertaken for ecotoxicological evaluation, where the knowledge of pollutant levels is of paramount importance. The compositional biplot conveys only relative information because of the compositional structure of the data, so that loadings cannot be interpreted separately, but only as pairs or groups of variables. Such grouping may make the overall picture less easy to understand. It is therefore strongly recommended to use compositional data analyses as a complement to the traditional approach (which consists in closely examining raw concentration levels in lichens, possibly also using concentration normalization), when mapping a synthetic contamination index, or for in-depth studies of the associations between variables, or between individuals.

## Conclusion

Our results demonstrate that epiphytic lichens, at least those belonging to the Parmeliaceae family, can be used as biomonitors for air quality assessment in a Ni mining context. As expected, raw Ni, Co, Cr concentrations in lichen bodies are high, even extremely high, close to the mine and to the ore treatment plant. Concentrations decrease as distance from these infrastructures increases. In our case, the sample scores for the first principal component, computed after appropriate transformation, can be used as an overall environmental indicator. The PC1 includes all the main elements emitted into the atmosphere during Ni extraction and treatment. This approach may provide valuable information for the management and protection of ecosystems, highlighting those areas most contaminated by mining activity, and identifying the best sites for the installation of costly filtration units, for continued air quality monitoring. However, as the synthetic index drawn from PC1 drastically simplifies the problem by providing a broad picture, it may also preclude the differentiation of sources, when their compositional differences are subtle. Applying compositional data analyses in combination with close examination of raw data from lichens is therefore recommended, because contact with their absolute values is lost during the statistical analysis. Although the results of this study indicate that lichens can be used beneficially for assessing atmospheric dispersion of Ni in the surroundings of mining and metallurgical activities, further study in an area where air quality or atmospheric deposition data are also available would be useful to evaluate more precisely the performances of the approach described here.
